# The Impact of Extrusion Cooking on the Physical Properties, Functional Components, and Pharmacological Activities of Natural Medicinal and Edible Plants: A Review

**DOI:** 10.3390/foods14111869

**Published:** 2025-05-24

**Authors:** Yao Xu, Fan Jia, Yuhang Wu, Jiani Jiang, Tao Zheng, Hui Zheng, Yong Yang

**Affiliations:** College of Pharmacy, Hunan University of Chinese Medicine, Changsha 410208, China; xuyao@stu.hnucm.edu.cn (Y.X.); jiafan1227@163.com (F.J.); 20233794@stu.hnucm.edu.cn (Y.W.); 20233795@stu.hnucm.edu.cn (J.J.); 004247@hnucm.edu.cn (T.Z.)

**Keywords:** extrusion cooking, natural medicinal and edible plants, functional components, pharmacological activities, physical properties

## Abstract

Extrusion cooking is an innovative, advanced processing technology widely used in the food and feed industries. With growing concerns over the health attributes of food, the effects of extrusion cooking on the functional characteristics of natural medicinal and edible plants (NMEPs) have attracted increasing attention from researchers. This review, based on recent literature on extrusion cooking, systematically summarizes its impact on the physical properties; functional components, such as total polyphenols, total flavonoids, total polysaccharides, and total saponins; and pharmacological activities, including antioxidant, hypoglycemic, hypolipidemic, and anti-inflammatory effects, of NMEPs. The aim is to provide a scientific basis for the application of extrusion cooking technology in the advanced processing of these resources.

## 1. Introduction

Extrusion cooking is a sophisticated thermal processing engineering technique that integrates multiple unit operations, including conveying, mixing, heating, pressurization, and shearing. This technology has been extensively applied in the processing of human food [1,2,3] and animal feed [4,5]. Because extrusion cooking combines multiple operations, it offers advantages in terms of efficiency, nutrient retention, and environmental friendliness over traditional thermal processing techniques.

Extrusion cooking is also commonly utilized as a pretreatment method for certain natural medicinal and edible plants (NMEPs), aiming to leverage the physicochemical modifications induced by the extrusion process. These modifications enhance the sensory properties, nutritional characteristics, and application functionalities of the raw materials. The principle of extrusion cooking involves subjecting the raw material to elevated temperatures and pressures within the extrusion die. The material then undergoes a rapid decompression from a high-temperature, high-pressure state to ambient conditions. This sudden pressure drop, coupled with rapid water evaporation, results in drying or puffing of raw materials [6].

Since extrusion cooking needs to maintain a high temperature and high-pressure environment in order to bring the puffing effect to the raw material, its extrusion temperature is usually 120–180 °C [7]. The moisture content of the raw material should not be too high, usually between 10% and 30% [7]. The operator can also adjust the temperature, pressure, feed rate and screw speed of the extrusion cooking machine to control the processing standard, respectively.

**Figure 1:**
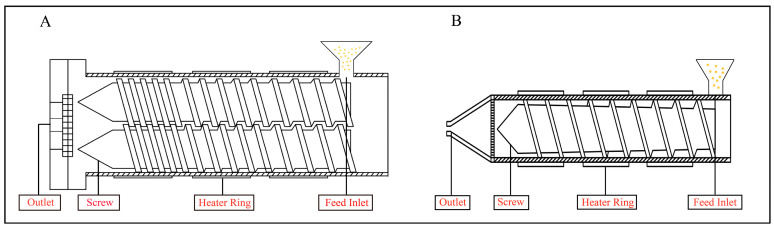
Schematic diagram of extruder: (**A**) twin-screw, (**B**) single screw.

As a high-temperature and high-pressure processing technology, extrusion cooking inevitably induces physicochemical effects that modify the physical properties, nutritional attributes, and functional activities of NMEPs. Currently, extrusion equipment is primarily categorized into two types: single-screw extruders and twin-screw extruders. Single-screw extruders are characterized by their low cost and operational simplicity [8]. However, they are associated with issues such as uneven material mixing and limited production capacity, making them suitable only for specific production environments. In contrast, twin-screw extruders offer advantages such as superior self-cleaning ability, stable material conveyance, and broader adaptability, making them the preferred choice in most production processes [9]. A schematic diagram of twin-screw (or single-screw) extruders can be found in Figure 1.

NMEPs refer to a category of edible or medicinal materials that confer health benefits to humans. The health-promoting functions of these resources are derived from their diverse functional chemical components, such as polysaccharides, flavonoids, saponins, and alkaloids [10,11]. These resources serve as the primary raw materials for traditional Chinese medicinal diets, healthy dietary regimens, functional foods, and herbal medicines. Through appropriate processing or rational formulation, NMEPs are utilized for dietary therapy, health maintenance, and disease treatment. Over time, conventional processing techniques, including cutting, drying, crushing, and extraction, have been extensively studied and understood. The effects of these techniques on the physicochemical properties and pharmacological health functions of NMEPs have been widely investigated. As a novel engineering technology, the application of extrusion cooking in the processing of NMEPs, along with its impact on their functional properties, remains in the exploratory research phase. Past reviews on extrusion cooking have generally focused only on chemical or physical changes in the raw materials, not on changes in their pharmacological activity. Unlike ordinary natural food resources, the functional characteristics of NMEPs, such as changes in functional components and health benefits, are the primary focus of processing technologies. With the increasing demand for functional extruded puffed foods and restructured extruded foods, understanding the influence of extrusion cooking on the functional properties of these resources is of practical significance for the scientific application of extrusion cooking technology in the field of healthy foods.

This review is based on recent literature reporting on the processing of NMEPs using extrusion cooking. It reviews the research progress on the effects of extrusion cooking on the physical properties, chemical characteristics, and health-promoting functions of these resources. The aim is to provide a reference for the application of this technology in the processing of medicinal and edible resources.

## 2. Effects of Extrusion Cooking on Physical Characteristics

### 2.1. Structural Characteristics

Extrusion cooking subjects raw materials to a high-temperature and high-pressure environment, leading to structural changes in the processed materials. Ren et al. [12] found that the microscopic surface structure of wheat bran transformed from dense and relatively smooth to a porous honeycomb structure after extrusion cooking. During extrusion, macromolecules in the raw materials degrade under high temperatures, pressures, and shear forces, resulting in reduced molecular size or altered polymerization states. Consequently, the post-extrusion material exhibits a loose and porous structure, with some extrudates expanding to 2–10 times their original volume [13]. Qi et al. [14] observed the microstructure of cassava flour before and after extrusion cooking using scanning electron microscopy and found that its ordered structure and rigid granular morphology were disrupted, transforming into a disordered and fragmented state. Chen et al. [15] reported that the natural crystalline structure of taro flour became disordered after extrusion cooking, producing crystalline fragments and increasing structural disorder. Wheat germ protein, with its high globulin and gluten content, has limited functionality in terms of solubility and emulsification, restricting its application in food processing. However, extrusion cooking improves its emulsifying properties by enhancing the emulsifying capacity of wheat germ albumin, globulin, and gliadin, significantly increasing the zeta potential of the emulsion and thereby improving its stability [16,17]. In summary, extrusion cooking can modify the structural characteristics of plant materials, and these changes may potentially influence their bioactivity.

### 2.2. Hydration Properties

The water absorption index (WAI) and water solubility index (WSI) are commonly used indicators to evaluate the hydration properties of products. WAI reflects the degree of starch gelatinization, and foods with higher WAI values induce a greater sense of satiety. WSI measures the degree of starch degradation, with higher WSI values indicating fewer macromolecules and easier digestibility [18]. Qiu et al. [19] found that WAI and WSI values of grains such as japonica rice, wheat, glutinous rice, millet, oats, rye, black rice, and black beans increased several to dozens of times after extrusion cooking. This was attributed to the increased content of soluble nutrients, particularly the enhanced solubility of gelatinized starch. Fang et al. [20] used extrusion cooking to prepare purple brown rice flour and analyzed changes in its physicochemical properties, finding that WAI and WSI increased by 1.07 and 2.80 times, respectively, after extrusion. The high mechanical shear force during extrusion disrupts the structural state of macromolecules. It exposes the hydrophilic groups in starch and protein, which promotes water absorption and improves hydration capacity [21].

**Figure 2:**
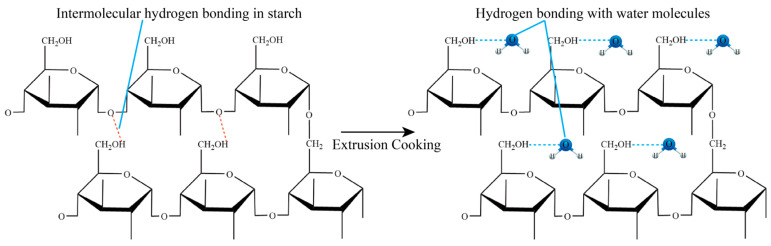
Principle of WAI value change during extrusion cooking (The red dotted line in the figure shows intramolecular hydrogen bonding, and the blue dotted line shows hydrogen bonding with water molecules).

Prince et al. [17] investigated the effect of extrusion cooking parameters on the physical and functional properties of intermediate wheatgrass. Screw speed was found to have a strong effect on WAI and WSI of intermediate wheatgrass. WAI reached a minimum value (4.5 g/g DM) at a screw speed of 200 rpm and WSI reached a maximum value (+20.92%) at a screw speed of 400 rpm. The increase in screw speed leads to a significant decrease in WAI and a significant increase in WSI, which is due to the fact that the higher the screw speed the stronger the shear force, the stronger the destructive force on the macromolecules, and the intermolecular hydrogen bonding is broken more seriously. The original intermolecular hydrogen bonding is transformed into hydrogen bonding with water molecules, and the hydrogen bonding with water molecules can effectively improve the hydration properties of raw materials (Figure 2).

In conclusion, extrusion cooking improves WAI and WSI to varying degrees, enhancing the nutritional content and physical properties of products. Extruded products thus offer better satiety and improved nutrient absorption.

### 2.3. Sensory Properties

The utilization of NMEPs in health applications is often constrained by their sensory characteristics. The taste and texture of raw materials directly influence consumer acceptance and the likelihood of long-term consumption. Extrusion cooking, as a technology for refining coarse grains, can effectively improve the sensory properties of food ingredients. Wang et al. [22] found that *Ginkgo biloba* L. became crispier and less bitter after extrusion cooking, while retaining its characteristic aroma and, to some extent, enhancing its nutritional value. *Chenopodium quinoa* Willd., which contains saponins in its outer shell that impart a strong bitter taste, traditionally has poor palatability. Li et al. [23] processed quinoa using extrusion cooking at 160 °C and quinoa flavor was determined using an electronic tongue. It was found that the bitter flavor of extrusion-cooked quinoa was reduced and the sweet flavor was increased. Extrusion cooking effectively improved the palatability of quinoa. Extrusion cooking effectively improved the palatability of *Chenopodium quinoa* Willd. Additionally, Li et al. [24] found that extrusion cooking kidney beans with the addition of sodium carbonate successfully eliminated the unpleasant smell of kidney beans. Analysis using headspace solid-phase microextraction coupled with gas chromatography-mass spectrometry revealed a reduction in volatile compounds such as aldehydes, alcohols, and aromatic hydrocarbons after extrusion cooking. Many plants are inherently coarse grains with traditionally poor sensory qualities, and extrusion cooking can significantly improve their sensory properties. At the same time, the impact of extrusion cooking on the functional natural components and health benefits of these materials is receiving increasing attention.

## 3. Effects of Extrusion Cooking on Bioactive Components

### 3.1. Functional Polysaccharides

Polysaccharides are high-molecular-weight natural polymers composed of more than ten monosaccharides linked by glycosidic bonds. They represent one of the most abundant biomass resources in nature. Functional polysaccharides, particularly non-starch polysaccharides, are key bioactive components in many NMEPs. Extrusion cooking can directly modify the molecular structure and aggregation state of most polysaccharides, thereby enhancing their solubility and improving their bioavailability for intestinal probiotics.

Studies have demonstrated that the polysaccharide content of *Dioscoreae Rhizoma* increases by 38.38% after extrusion cooking [25], while the oat polysaccharide content in oat bran increases by 81.86% [26]. Polysaccharides extracted from extruded oat bran exhibit significantly improved solubility, transparency, oil absorption capacity, and solvent retention capacity compared to non-extruded raw materials, although their thermal stability is reduced. *Pinelliae Rhizoma* polysaccharides are the primary bioactive components responsible for the antitumor effects of *Pinelliae Rhizoma*. Research has shown that extrusion cooking significantly increases the polysaccharide content of *Pinelliae Rhizoma*, suggesting that this process may enhance its antitumor efficacy [27]. Other NMEPs, such as *Ganoderma* [28], *Phellinus igniarius* [29], *Hericium erinaceus Pers.* [30], and *Inonotus obliquus* [31], also exhibit increased polysaccharide content after extrusion cooking. Table 1 summarizes the changes in polysaccharide content of NMEPs after extrusion cooking.

The expansion effect induced by extrusion cooking significantly increases the specific surface area of raw materials. Simultaneously, the high temperature and pressure conditions disrupt the aggregation state of polysaccharide molecules and cause molecular degradation. In addition, the high shear force generated during extrusion cooking produces a substantial cell wall disruption effect. The combined effects of these mechanisms enhance the dissolution rate of polysaccharides, primarily manifested as an increase in polysaccharide content in the raw materials.

### 3.2. Polyphenolic Compounds

Polyphenols, a broad class of phenolic derivatives, are natural phytochemicals with diverse pharmacological activities, including antioxidant, anti-inflammatory, and antitumor effects. As the most abundant dietary antioxidants, their stability during storage and processing is critical for preserving functional properties. Zhang et al. [35] demonstrated that extrusion cooking significantly increased total flavonoids (TFC) in *Rhodiolae Crenulatae Radix Et Rhizoma* from 6.42 mg/g to 11.75 mg/g, representing an 83% enhancement. Conversely, Zhang et al. [36] observed an 82.58% reduction in bound polyphenols but a 232.26% increase in free polyphenols in extruded brown rice, likely due to shear-induced conversion of bound phenolics into free forms [37]. These changes are summarized in Table 2.

The structural reorganization induced by extrusion—characterized by cell wall rupture and tissue loosening—enhances solvent penetration efficiency, thereby promoting polyphenol release [38]. Simultaneously, intense shear forces hydrolyze bound polyphenols into soluble free forms, amplifying extractable phenolic content. However, thermal degradation under high-temperature conditions may partially offset these gains by oxidizing heat-sensitive polyphenols [39]. Thus, the net change in polyphenol content reflects a balance between mechanical liberation and thermal degradation.

Table 2 shows the changes in TFC and total polyphenols content (TPC) of some NMEPs after extrusion cooking. As can be seen from Table 2, most of the TPC and TFC decreased after extrusion cooking of cereals. But most of the TPC and TFC increased after extrusion cooking of Chinese herbs. Suggesting that extrusion cooking is beneficial to polyphenol solubilization of Chinese herbs. It was hypothesized that the reason for this might be related to the differences in the microstructure of the raw materials. Grain and cereals are mostly endosperm structures, and their main component is starch. The internal functional components are fewer, and the dissolution effect of the extrusion cooking process is smaller. On the contrary, the high temperature and high-pressure environment further reduces the polyphenols that are already in small quantities. The raw materials of Chinese herbal medicine are mostly cellular structures, often with special storage structures or thick-walled cells for the accumulation of active ingredients. For example, the bast of Astragalus contains a large number of bound polyphenols, which are released in large quantities after extrusion cooking and processing [40].

The changes in the TPC of NMEPs after extrusion cooking are not only related to the characteristics of the raw material, but the extrusion cooking temperature is also a key influencing factor. Most of the NMEPs were extruded at temperatures lower than 150 °C, such as *quinoa* [23], *Dioscoreae rhizoma* [25], *Phellinus igniarius* [29], *White ginseng* [34], *Rhodiolae crenulatae radix et rhizoma* [35], and *Astragali radix* [40]. Extrusion cooking at less than 150 °C can effectively reduce the loss of polyphenolic compounds.

**Table 2:** Changes in TFC and TPC of NMEPs after extrusion cooking.

Source	Content in Input Raw Material (mg/g DM)		Content in Output Extrudates (%)		References
	TFC	TPC	TFC	TPC	
*Barley*	—	3.0 ~ 4.4	—	−12.0~30.0	[1]
*Purple brown rice*	3.9	2.8	−28.3	−23.7	[20]
*Quinoa*	3.7	2.2	−27.4	−11.0	[23]
*Dioscoreae rhizoma*	—	1.6	—	+4.2	[25]
*Phellinus igniarius*	28.1	14.0	+60.4	+73.4	[29]
*White ginseng*	—	2.7	—	+31.4~202.5	[34]
*Rhodiolae crenulatae radix et rhizoma*	6.4	18.3	+218.3	+38.4~83	[35]
*Astragali radix*	3.1	—	+3.1~5.3	—	[40]
*Polygonati odorati rhizoma*	5.2	—	+69.4	—	[41]
*Highland barley*	—	1.6	—	−49.5	[42]
*Buckwheat*	5.4	10.0	−39.7	−36.8	[43]
*Apios americana Medik*	2.9	1.8	+20.9	+12.9	[44]
*Wheat germ*	10.5	3.4	−2.7	−11.1	[45]

Note: “—” indicates that the value was not measured.

### 3.3. Saponins

**Figure 3:**
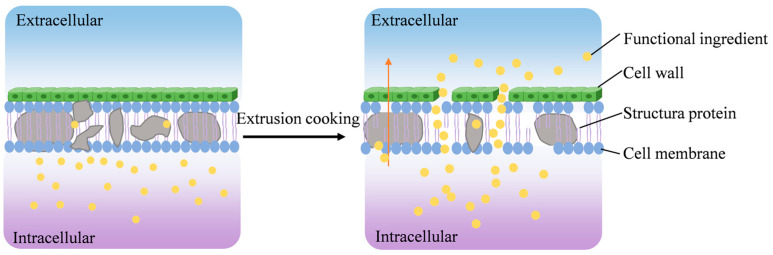
Principle of functional component change.

Plant saponins exhibit various pharmacological activities, including antimicrobial, antioxidant, and immune-modulating properties. The processing stability of saponins NMEPs is closely related to the moisture content of the raw materials and processing temperature. Yan et al. [46] found that the total saponin content (TSC) in ginseng and white ginseng gradually decreased with increasing extrusion cooking temperature. The highest TSC content (7.31%) was achieved at a moisture content of 25% and an extrusion cooking temperature of 110 °C. Jin et al. [47] prepared rice wine using a mixture of rice and ginseng that had undergone co-extrusion cooking. The TSC content of ginseng in the rice wine significantly increased, and the sensory evaluation of the wine was improved. Cao et al. [48] reported that the TSC content of *Polygonati rhizoma* increased substantially after extrusion cooking, with an increase of up to 100%. Extrusion cooking can also be utilized to eliminate saponin-related antinutritional factors and bitter compounds. For example, saponins in quinoa hulls impart a strong bitter taste. Kowalski et al. [49] observed a 97.31% reduction in quinoa saponins after extrusion cooking, effectively eliminating the bitterness. The mechanisms behind these changes in saponin content are likely the cell wall disruption and hydrolysis effects induced by extrusion cooking (Figure 3). In practical applications, processing objectives should be clearly defined to leverage the advantages of extrusion cooking for purposes such as enhancing processing efficiency or improving flavor.

### 3.4. Terpenoids

Terpenoids are a class of compounds with the general formula (C_5_H_8_)n, including their oxygenated and variously saturated derivatives. Naturally occurring terpenoids exhibit a wide range of pharmacological activities, such as anti-inflammatory, immune-modulating, and antitumor effects. Terpenoids are important functional components in many NMEPs. Cueto et al. [50] used corn mixtures subjected to extrusion cooking at a moisture content of 14% and a temperature of 150 °C. It was found that the content of terpenoids in classified carotenoids increased significantly after extrusion cooking compared to the non-extruded raw material, probably due to the wall-breaking effect of extrusion cooking that promotes the migration and leaching of lipids from the cells. Ortiz et al. [51] found that carotenoids retention in extruded cooked maize at 35% moisture content was 94.2%, whereas the carotenoids retention in extruded cooked maize was only 65% at 25% moisture content, which suggests that high moisture extrusion cooking is more conducive to the processing stability of carotenoid than low moisture extrusion cooking.

It was also found that lutein and zeaxanthin decreased after extrusion cooking of intermediate supine wheat [47]. The extrusion cooking parameter, screw speed, was the key influencing factor. The retention of lutein was 24.2% (200 rpm) and 33.7% (300 rpm), and that of zeaxanthin was 34.2% (200 rpm) and 49.6% (300 rpm). This may be due to the fact that the gain in solubilization effect from extrusion cooking at high rotational speeds is higher than the destruction of terpenoids by shear. This resulted in a lower rate of loss of lutein and zeaxanthin from NMEPs at higher screw speeds.

### 3.5. Anti-Nutritional Factors

Common anti-nutritional factors in NMEPs include phytic acid, tannins, and enzyme inhibitors. The high-temperature and high-pressure effects of extrusion cooking can effectively reduce the content of these anti-nutritional factors, thereby improving the nutritional value and safety of food products. Table 3 summarizes changes in anti-nutritional factors in selected NMEPs after extrusion cooking. Rahul et al. [52] reported that tannins, phytic acid, and trypsin inhibitor content in lentil splits decreased by 98.83%, 99.30%, and 99.54%, respectively, after extrusion cooking. Marzo et al. [53] found that extrusion puffing significantly reduced the levels of phytic acid and tannins in kidney beans, along with a notable decrease in the activity of lectins, trypsin, chymotrypsin, and α-amylase. Rats fed with extruded kidney beans exhibited significantly improved growth rates, while those fed raw kidney beans mostly died by the ninth day. Additionally, it has been observed that feeding pigs extruded rapeseed effectively enhances nutrient digestibility and growth performance [54]. Extrusion cooking reduces the activity or content of anti-nutritional factors in NMEPs, thereby improving the bioavailability of protein and minerals in animals. Obiang-Obounou et al. [55] demonstrated that extrusion cooking significantly reduced tannin content in chestnuts. Compared to non-extruded chestnuts, tannin content decreased by 78% when extrusion cooking was conducted at 120 °C and 25% moisture content. Extrusion cooking is highly effective in eliminating heat-labile anti-nutritional factors [56], but its efficacy is limited for heat-stable anti-nutritional factors such as alkaloids [57].

### 3.6. Other Effects

After primary processing of NMEPs, a significant portion of by-products is often generated, which are commonly utilized in the production of animal feed. However, some NMEPs by-products may contain harmful substances that negatively impact animal growth performance and health. Conventional processing methods are often ineffective in degrading these harmful substances, or their implementation is cost-prohibitive for large-scale production.

Deng [63] demonstrated that extrusion cooking of cottonseed meal reduced gossypol toxicity by 63%. When broilers were fed with extruded cottonseed meal, their growth performance significantly improved, and the activities of alanine aminotransferase and aspartate aminotransferase in the broilers were significantly higher compared to those fed non-extruded cottonseed meal, indicating improved safety and nutritional value of the extruded product. Furthermore, studies have shown that extrusion cooking can reduce fumonisin levels in corn gluten meal by 90% [64] and aflatoxin B1 levels in peanut meal by 77% [65].

The application of extrusion cooking in animal feed production not only effectively degrades specific toxins in raw materials but also enhances the overall utilization of plant by-products while maintaining sensory qualities. This approach provides a cost-effective and practical solution for improving the safety and nutritional value of animal feed.

## 4. Effects of Extrusion Cooking on Pharmacological and Health-Promoting Activities

### 4.1. Adsorption Activity of Harmful Ions

Dietary fiber (DF), composed of soluble dietary fiber (SDF) and insoluble dietary fiber (IDF), plays a significant role in nutrition and functionality, with SDF exhibiting greater physiological effects. Zhang et al. [66] demonstrated that extrusion cooking increases the SDF content in oat bran, enhancing its solubility, swelling capacity, and solvent retention ability. Similarly, DF extracted from peanut shells after extrusion cooking showed significantly improved adsorption capacity for heavy metal ions such as Pt^2+^ (239.5 ± 2.5 μmol·g^−1^ → 378.5 ± 5.3 μmol·g^−1^), Cu^2+^ (100.4 ± 3.1 μmol·g^−1^ → 167.2 ± 2.5 μmol·g^−1^), and As^3+^ (198.5 ± 2.9 μmol·g^−1^ → 278.3 ± 3.2 μmol·g^−1^) [67]. This enhancement is attributed to the increased specific surface area of peanut shells after extrusion cooking, which facilitates greater contact with metal ions. Extrusion cooking also tripled the Cu^2+^ adsorption capacity of soybean DF compared to untreated raw materials [68]. Moreover, DF extracted from enzyme-assisted extruded foxtail millet exhibited a fivefold increase in NO^2−^ adsorption and retention capacity compared to non-extruded millet [69]. Enhanced NO^2−^ adsorption was also observed in extruded bamboo shoots [70] and broad bean-konjac mixed flour [71]. The improved ion adsorption capacity of extruded materials is likely due to reduced particle size, increased molecular surface area, and the exposure of functional chemical groups. Additionally, extrusion cooking can convert some IDF into SDF, while the high temperature, pressure, and shear forces disrupt glycosidic bonds and intramolecular hydrogen bonds, enhancing solubility and physiological activity [72]. The changes in functional activity of some NMEPs after extrusion steaming can be seen in Table 4.

### 4.2. Lipid Regulation Activity

Hyperlipidemia, a metabolic disorder characterized by abnormal blood lipid levels, is a high-risk factor for many metabolic diseases. Promoting intestinal cholesterol excretion can lower serum cholesterol levels, thereby regulating blood lipids [73]. Wang et al. [74] found that rats fed extruded *Highland Barley* exhibited significantly higher serum HDL-C levels and lower LDL-C levels compared to those fed non-extruded *Highland Barley*. The enhanced lipid-regulating activity of extruded *Highland Barley* may be attributed to the reduced molecular weight of β-glucan [75] and improved utilization by gut microbiota [76].

Ding et al. [71] reported that extrusion cooking significantly increases the in vitro adsorption capacity of a mixed flour (broad bean, buckwheat, and konjac) for bile salts and cholesterol. Similarly, Song et al. [70] observed that extruded bamboo shoot DF, after the removal of protein and starch, exhibited superior cholesterol adsorption capacity (8.6 mg/g at pH 7) compared to non-extruded DF (5.7 mg/g at pH 7). Improved cholesterol adsorption was also noted in extruded rice bran [77] and orange pomace [78] under simulated intestinal conditions. The mechanisms underlying the enhanced lipid-regulating activity of extruded plant materials include the degradation and functional enhancement of active polysaccharides and increased cholesterol adsorption capacity.

### 4.3. Hypoglycemic Activity

Traditional medicinal and edible plants, such as *Dioscoreae rhizoma*, *Polygonati odorati rhizoma*, and *Poria*, as well as coarse grains like oats, buckwheat, and quinoa, are widely used as functional ingredients in health food manufacturing to achieve blood sugar control. Extrusion cooking technology significantly enhances the palatability of these raw materials, improving consumer acceptance and sustainability of health management. Yang et al. [79] reported that extrusion cooking enabled the preparation of low-glycemic index foods with excellent palatability using a combination of *Barley*, black rice, and quinoa. Miehle et al. [80] found that extruded citrus dough exhibited significantly lower in vitro glucose release (1170 mg/dL) compared to non-extruded dough (1462 mg/dL). Liang et al. [81] demonstrated that extruded *Rosa Roxburghii Tratt* was more effective in regulating metabolic parameters and alleviating diabetic symptoms in diabetic mice than non-extruded raw materials. Similarly, Muhammad et al. [82] observed that extruded oat bran exhibited stronger hypoglycemic effects in normal, hypercholesterolemic, and diabetic rats compared to raw oat bran.

**Table 4:** Changes of extrusion cooking on the functional activity of NMEPs and optimal processing conditions.

Source	Changes in Health-Promoting Activities	Processing Condition	References
*Oat bran*	Swelling capacity: 1.45 to 2.02 mL·g^−1^. Solvent retention capacity: 0.86 to 1.20 mL·g^−1^.	Temperature: 140 °C Screw speed: 150 rpm Moisture content: 10%	[66]
*Peanut shell*	Adsorption capacity for Pt^2+^, AS^3+^, and Cu^2+^: from 239.5 ± 2.5, 198.5 ± 2.9, and 100.4 ± 3.1 μmol·g^−1^ to 378.5 ± 5.3, 278.3 ± 3.2, and 167.2 ± 2.5 μmol·g^−1^.	Temperature: 130 °C Screw speed: 200 rpm Moisture content: 20%	[67]
*Soybean*	Adsorption capacity for Pt^2+^, AS^3+^, and Cu^2+^ 3: from 60.3, 52.9, and 32.7 μmol·g^−1^ to 212.4, 192.1, and 121.3 μmol·g^−1^.	Temperature: 150 °C Screw speed: 180 rpm Moisture content: 17%	[68]
*Millet*	Cholesterol adsorption capacity and nitrite ion adsorption capacity were increased.	Temperature: 190 °C Screw speed: 30 Hz	[69]
*Bamboo shoot*	Nitrite ion adsorption capacity: 24.1 to 26.5 µg/g. Glucose adsorption capacity: 100 to 122 mg·g^−1^. Cholesterol adsorption capacity: 3.3 to 7.5 mg·g^−1^	No detailed conditions	[70]
*Broad bean-konjac*	Cholesterol adsorption capacity: 4.9 to 5.5 μmol·g^−1^. Nitrite ion adsorption capacity: 421.7 μg·g^−1^.	Temperature: 150 °C Screw speed: 150 rpm Moisture content: 16%	[71]
*Hulless barley*	Total cholesterol: 2.77 to 1.70 mmol·L^−1^. LDL-C: 0.14 To 0.12 mmol·L^−1^.	Temperature: 160 °C Screw speed: 45 rpm	[74]
*Rice bran*	Glucose binding capacity: 325 to 460 mg·g^−1^. Bile salt binding capacity: 25.10 to 65.52 mg·g^−1^. Cholesterol binding capacity: 2.10 to 2.60 mg·g^−1^.	Temperature: 120 °C Screw speed: 250 rpm Moisture content: 17%	[77]
*Orange pomace*	Glucose binding capacity: 721 to 752 μmol·g^−1^. Cholesterol binding capacity: 6.89 to 11.92 mg·g^−1^. Bile acid binding capacity: 38.5% to 61.1%.	Temperature: 129 °C Screw speed: 299 rpm Moisture content: 15%	[78]
*Citrus fiber*	In vitro glucose release: 1462 mg/dL to 1170 mg/dL.	Temperature: 150 °C	[80]
*Rosa roxbunghii pomace*	Blood glucose values were 36.21% and 59.98% lower than the model group at days 7 and 14, respectively.	Temperature: 150 °C Screw speed: 144 rpm Moisture content: 33%	[81]

The improved blood sugar control after extrusion cooking is primarily attributed to the increased content of SDF and changes in physicochemical structure. The high viscosity of SDF delays glucose absorption in the gastrointestinal tract. Additionally, structural modifications such as reduced particle size, increased surface area, and the formation of porous fiber networks induced by extrusion cooking further impede glucose absorption [83].

### 4.4. Antioxidant Activity

Excessive free radicals in the body can cause oxidative damage to lipids, proteins, and nucleic acids, accelerating cellular aging [84]. Plants contain abundant antioxidants that can effectively neutralize excess free radicals, reduce oxidative damage, and delay aging. Therefore, the impact of processing on the antioxidant activity of plants has garnered significant attention. Zhang et al. [35] found that extrusion cooking enhanced the DPPH and ABTS radical scavenging activities and total reducing power of *Rhodiolae Crenulatae Radix Et Rhizoma*. Agnieszka et al. [85] reported that extrusion cooking increased the DPPH radical scavenging capacity of a *Lycii fructus* composite (rice flour, wheat flour, and *Lycii fructus*) by 100% and ABTS radical scavenging capacity by 60.8%, likely due to an 84% increase in total phenolic content. This increase may result from the mechanical effects of extrusion cooking, which degrade cell walls and promote the release of bound phenolics [86].

However, extrusion cooking can also reduce the antioxidant capacity of certain plants. Ashrafi et al. [87] observed that extrusion cooking decreased the DPPH radical scavenging activity and total antioxidant activity of maize by 39–43% and 42–57%, respectively, accompanied by a 28–35% reduction in total phenolics and a 30–37% reduction in flavonoid content. Similar reductions in antioxidant activity have been reported for purple brown rice [20], white rice [36], and blackcurrant pomace [88].

The high temperature and pressure of extrusion cooking can degrade and oxidize heat-sensitive phenolic compounds, reducing antioxidant capacity [89]. Conversely, the shear and cell wall disruption effects of extrusion cooking can convert bound phenolics to free phenolics, enhancing antioxidant activity. The overall impact of extrusion cooking on antioxidant activity depends on the balance between these opposing effects, which vary with extrusion methods, processing conditions, and raw material types. Therefore, selecting appropriate processing parameters is crucial for specific raw materials. The effects of extrusion cooking on the antioxidant activity of selected NMEPs are summarized in Table 5.

### 4.5. Anti-Inflammatory Activity

Inflammation is a defense mechanism triggered by the body to eliminate invading pathogens. While moderate inflammation can eradicate pathogenic factors, excessive inflammation leads to oxidative stress [91]. Under oxidative stress, tissues are influenced by inducible nitric oxide synthase (iNOS), resulting in a rapid increase in NO production, which exacerbates inflammatory responses. Studies have confirmed that suppressing NO production in tissues can alleviate and control inflammation. Stefano et al. [92] found that extrusion cooking enhanced the anti-inflammatory effects of germinated bean cotyledons. This improvement was attributed to the increased release of anti-inflammatory bioactive peptides after extrusion, which enhanced NO inhibitory activity in vivo and reduced the phosphorylation of inflammation-related pathway proteins. Leem et al. [93] demonstrated that extruded Acanthopanax senticosus leaves exhibited significantly stronger inhibition of inflammatory cytokine expression compared to non-extruded raw materials. Specifically, the extracts from extruded leaves showed 1.5-fold, 2.0-fold, and 1.2-fold greater inhibition of MCP-1, TNF-α, and IL-1β, respectively. Additionally, the concentrations of key anti-inflammatory compounds, such as elutheroside-E and chiisanoside, increased 2- to 10-fold after extrusion [94].

Tumor necrosis factor (TNF) and interleukins (ILs) play critical roles in inflammation by activating multiple signaling pathways, such as nuclear factor-kappa B (NF-κB), thereby promoting the production of inflammatory factors. Alvaro et al. [95] used LPS-induced human THP-1 cells and murine RAW 264.7 macrophages as experimental models to compare the anti-inflammatory effects of extruded and non-extruded amaranth protein hydrolysates. The extruded hydrolysates exhibited superior anti-inflammatory effects, reducing TNF-α levels by 36.5% and 33.5%, PGE2 levels by 15.4% and 31.4%, and COX-2 levels by 38.1% and 67.6% in THP-1 cells and RAW 264.7 macrophages, respectively. Qiu et al. [96] investigated the anti-inflammatory effects of extruded black rice extracts on LPS-stimulated RAW 264.7 cells and found enhanced activity. This was attributed to a 3.11-fold increase in protocatechuic acid content in the extruded extracts compared to non-extruded raw materials. Extrusion cooking disrupts cell wall structures through mechanical shear forces, facilitating the release of bioactive compounds. The application of extrusion cooking to specific plant materials can effectively enhance the release of functional components and improve their health benefits.

### 4.6. Immunomodulatory Activity

β-Conglycinin, a major component of soybean 7S globulin, is a key antigenic protein responsible for soybean allergies. Reducing the antigenicity of soybeans is essential for improving food safety. Extrusion cooking can reduce the antigenicity of legume materials while enhancing their sensory quality. Yin et al. [97] found that extrusion cooking significantly decreased the antigenicity of β-conglycinin, with antigenicity decreasing as extrusion temperature and screw speed increased and feed rate decreased. Chang et al. [98] used immunoblotting to analyze the binding capacity of extruded soybean proteins to epitope antibodies. They observed that extruded proteins showed significantly reduced binding to five out of ten epitope antibodies compared to non-extruded proteins, indicating that extrusion cooking disrupts certain antigenic epitopes, thereby lowering antigenicity.

Immunomodulatory peptides are biologically active peptides that enhance immune function by promoting lymphocyte proliferation and macrophage phagocytosis. They also boost immune resistance to pathogen infections, reducing disease incidence [99]. Soybean bioactive peptides, derived from the enzymatic hydrolysis of soybean proteins, are more efficiently extracted after extrusion cooking. Compared to traditional wet-heat processing, extrusion increased the extraction yield of immunomodulatory peptides by 40% [100]. Zhang et al. [101] studied the effects of trypsin-hydrolyzed extruded soybean meal on splenic lymphocyte proliferation in mice and found that extruded hydrolysates enhanced lymphocyte proliferation compared to non-extruded raw materials. Extrusion cooking effectively improves the extraction yield and content of protein peptides from soybean materials, thereby enhancing their health effects. These findings provide new insights into the efficient production of immunomodulatory peptides using extrusion cooking technology.

## 5. Methods

### 5.1. Literature Search Methodology

Primary scientific databases, including Web of Science, PubMed, and CNKI, were queried using keywords such as the following: “extrusion cooking and physical properties”, “extrusion cooking and chemical composition”, “extrusion cooking and pharmacological effects”, and “extrusion cooking and plants”. Restricting the publication date from January 2000 to December 2024. The percentage of data cited in this study for the last 5 years is 41.7%, and for the last 10 years is 83.5%.

### 5.2. Limitations

While this review synthesizes key findings from the existing literature, several limitations should be acknowledged. First, the non-systematic nature of the literature search may have introduced selection bias, as relevant studies outside mainstream databases or in non-English languages might have been overlooked. Second, methodological heterogeneity across included studies—such as variations in sample sizes, experimental designs, and outcome measurement tools—limits direct comparability and meta-analytic synthesis. Third, publication bias likely skewed the evidence base toward statistically significant or positive results, potentially underrepresenting null or contradictory findings. Additionally, the rapid evolution of research in this field means some recent studies may not have been incorporated due to time constraints. These factors caution against overgeneralization of conclusions and highlight the need for future systematic reviews with rigorous inclusion criteria and quantitative bias assessments.

## 6. Discussion and Outlook

Extrusion cooking can improve the nutritional quality and physicochemical properties of NMEPs, and the changes in these aspects can be more deeply reflected in the pharmacological and healthcare activities. After extrusion cooking, NMEPs puff up into a loose porous structure. More IDF in NMEPs is converted to SDF, and it has been shown that SDF is more effective than IDF in many beneficial properties [102]. Due to the conversion of DF from insoluble to soluble in NMEPs, its solubility, swelling capacity, and solvent retention capacity are enhanced, and polysaccharides, which are less effective in the intestinal tract, can be more potent. At the same time, extrusion cooking prompted more polysaccharides to be dissolved, and the pharmacological activity of NMEPs was also enhanced after the polysaccharide extraction rate was increased. After extrusion and cooking, the adsorption capacity of the raw material to metal ions, cholesterol, and monosaccharides increased. It is speculated that extrusion cooking may potentially enhance the biological activity of NMEPs, such as anti-hypoglycemia, anti-hypolipidemia, and reduce the absorption of harmful metal ions

Extrusion cooking can destroy the plant cell wall structure and increase the release of bound polyphenols, which are bound within the cells and difficult to extract. Bound polyphenols are also often combined with cell wall polysaccharides or proteins through ester bonds and hydrogen bonds to form a bound state. Extrusion cooking further converts a large number of polyphenols from the bound state to the free state. Compared with the general heat processing method, the high-temperature and short-time processing characteristics of extrusion cooking also facilitate rapid deactivation of enzyme activity and reduction of polyphenol oxidation. As for terpenoids, they are more sensitive to temperature, and most of them will be lost during processing. Extrusion cooking is not recommended for NMEPs with high terpene content, or low temperature is recommended as a processing condition.

In summary, the effects of extrusion cooking on the physicochemical properties and pharmacological activities of NMEPs are interrelated. Alterations in physicochemical properties also potentially influence pharmacological activities. Therefore, future research should simultaneously examine the effects of extrusion cooking on both aspects and elucidate the relationship between physicochemical properties and pharmacological activities. A deeper study could have significant implications for the treatment of related diseases by extrusion cooking.

Health is the central theme of future food development, with unprecedented attention being paid to personal health issues. As foundational raw materials for the health industry, NMEPs are increasingly influenced by novel processing technologies in terms of their health-promoting factors and functionalities. To scientifically and rationally apply extrusion cooking technology at various stages of NMEPs processing, the following issues need to be addressed:

(1) The influence of extrusion cooking on different functional components needs to be further studied. Due to the complexity of NMEPs, the chemical changes of functional components under high-temperature and high-pressure conditions are also affected by many factors. Therefore, the results of extrusion cooking of NMEPs cannot be simply referred to and need to be analyzed from a variety of processing factors.

(2) In the extrusion cooking process, the processing conditions should be selected according to the actual purpose. For the two purposes of reducing the content of toxic and hazardous substances and increasing the extraction rate of functional substances, the selection of their processing conditions is completely different.

(3) Extrusion cooking technology can further affect the pharmacological health functions of NMEPs by affecting their chemical composition. There is a lack of in-depth studies on the relationship between these two changes. It would be beneficial to the field of extrusion cooking processing if subsequent studies could delve deeper into the mechanisms behind them.

## 7. Conclusions

Extrusion cooking induces multifaceted modifications in the physicochemical properties of NMEPs, including alterations in physical microstructure, chemical composition, and biofunctional attributes. During this thermomechanical process, thermally labile compounds undergo simultaneous degradation and polymerization, leading to dynamic fluctuations in the concentrations of bioactive constituents. The extraction rate of more functional components can be improved by disrupting the cell wall matrix and the binding between components during extrusion. For different NMEPs, different processing conditions need to be selected according to their botanical and processing properties. Choosing the right processing conditions of extrusion cooking can amplify the health-promoting potential of NMEPs.

## Figures and Tables

**Table 1:** Changes in polysaccharide content of NMEPs after extrusion cooking.

Source	Content in Input Raw Material (mg/g DM)	Content in Output Extrudates (mg/g DM)	Content Change (%)	References
*Red ginseng*	73.3	80.6	+9.9	[32]
*Dioscoreae rhizoma*	27.9	38.6	+38.3	[25]
*Oat bran*	89.7	163.1	+81.8	[26]
*Pinelliae rhizoma*	128.8	145.4	+12.8	[27]
*Ganoderma*	41.1	79.1	+91.7	[28]
*Phellinus igniarius*	0.7	2.5	+257.1	[29]
*Hericium erinaceus pers*	1.5	3.6	+140.0	[30]
*Inonotus obliquus*	61.9	88.5	+42.9	[31]
*Poria*	51.7	79.1~82.2	+52.9~58.9	[33]
*White ginseng*	40.3	49.9~77.2	+23.8~92.5	[34]

**Table 3:** Changes in anti-nutritional factors of some NMEPs after extrusion cooking.

Source	Types of Anti-Nutritional Factors	Content Change (%)	References
*Lentil splits*	phytic acid	−99.3	[52]
	tannins	−98.8	
	trypsin inhibitors	−99.5	
*Chestnuts*	tannins	−78.0	[55]
*Cereal brans*	phytic acid	−36.8	[58]
	oxalic acid	−54.5	
	trypsin inhibitors	−72.3	
*Kidney beans*	phytic acid	−26.8	[59]
	tannins	−55.4	
	agglutinin	−	
	protease inhibitor	−	
*Cowpea*	phytic acid	−33.2	[60]
	agglutinin	−	
	amylase inhibitor	−	
	trypsin inhibitors	−38.2	
*Kidney bean*	phytic acid	−28.0	[61]
	tannins	−68.9	
	protease inhibitor	−	
	agglutinin	−	
*Linseed*	tannins	−61.2	[62]

Note: “−“ indicates that the substance has been completely inactivated.

**Table 5:** Changes in Antioxidant Activity of some NMEPs after extrusion cooking.

Source	DPPH (%)	ABTS (%)	·OH (%)	Other Methods (%)	References
*Barley*	+33	—	—	FRAP − 22	[1]
*Purple brown rice*	+23.92	−20.1	−24.3	—	[20]
*Quinoa*	—	+5.9	−3.8	—	[23]
*Dioscoreae rhizoma*	—	+38.4	—	—	[25]
*White ginseng*	+202.5	—	—	FRAP + 30.4	[34]
*Rhodiolae crenulatae radix et rhizoma*	+31.2	+12.83	—	FRAP + 23.2	[35]
*Refined white rice*	—	—	−56.6	ORAC − 56.2	[36]
*White brown rice*	—	—	−52.3	ORAC − 56.8	[36]
*Astragali radix*	+16.7	—	+27.9	FRAP + 132.8	[40]
*Polygonati odorati rhizoma*	−45.5	—	−24.7	—	[41]
*Highland barley*	—	—	−25.3	FRAP − 9.8	[42]
*Buckwheat*	−49.4	−35.1	—	—	[43]
*Apios americana Medik*	+8.5	+21.0	—	—	[44]
*Lycii fructus*	+100	+60.8	—	FRAP + 84.3	[85]
*Maize*	−43	—	—	FRAP − 57 ORAC − 300	[87]
*Sprouted brown rice*	+60	—	−12.26	—	[90]
*Brown rice*	+105.3	+55.1	—	ORAC + 153.9	[42]

Note: —Indicates that the antioxidant value of the raw material was not determined using this method.

## Data Availability

No new data were created or analyzed in this study. Data sharing is not applicable to this article.

## Abbreviations

The following abbreviations are used in this manuscript:

Abbreviation	Definition
NMEPs	Natural Medicinal and Edible Plants
WAI	Water Absorption Index
WSI	Water Solubility Index
TSC	Total Saponin Content
TPC	Total Polyphenols Content
TFC	Total Flavonoids Content
IDF	Insoluble Dietary Fiber
DF	Dietary Fiber
SDF	Soluble Dietary Fiber
DPPH	1,1-Diphenyl-2-Picrylhydrazyl Radical Scavenging Activity
ABTS	2,2′-Azinobis-(3-Ethylbenzthiazoline-6-Sulphonate) Radical Scavenging Activity
FRAP	Ferric Reducing Antioxidant Power
ORAC	Oxygen Radical Absorbance Capacity
·OH	Hydroxyl Radical Scavenging Activity
